# Evolving landscape of glioblastoma research: A 75-year bibliometric study on survival, treatment and gender in authorship

**DOI:** 10.1007/s00508-026-02725-9

**Published:** 2026-03-10

**Authors:** Jana Breu, Bernhard Baumann, Jason K. Sa, Anna Sophie Berghoff, Matthias Preusser, Karl-Heinz Nenning, Adelheid Woehrer

**Affiliations:** 1 Division of Neuropathology and Neurochemistry, Department of Neurology, Medical University of Vienna, Vienna, Austria; 2 Center for Medical Physics and Biomedical Engineering, Medical University of Vienna, Vienna, Austria; 3 Institute of Biomedical Physics, Medical University of Innsbruck, Innsbruck, Austria; 4 Department of Biomedical Informatics, Korea University College of Medicine, Seoul, Korea (Republic of); 5 Department of Biomedical Sciences, Korea University College of Medicine, Seoul, Korea (Republic of); 6 Division of Oncology, Department of Medicine I, Medical University of Vienna, Vienna, Austria; 7 Center for Biomedical Imaging and Neuromodulation, Nathan Kline Institute, Orangeburg, NY USA; 8 Institute of Pathology, Neuropathology & Molecular Pathology, Medical University of Innsbruck, Innsbruck, Austria; 9 Muellerstraße 44, 6020 Innsbruck, Austria

**Keywords:** Neurooncology, Survival trends, Tumor treating fields, Sex disparities in authorship, Treatment outcomes

## Abstract

**Background:**

Glioblastoma is the most aggressive type of brain tumor. Here, we examine the evolution of glioblastoma treatment and survival across different study types over nearly a century. Furthermore, we map the landscape of glioblastoma research to track the potential influence of publication bias on perceived progress and assess sex disparities in authorship across time, study type and geographic region.

**Methods:**

We analyzed a comprehensive dataset of glioblastoma research published from 1947–2022. A manual review of 19,668 articles yielded 2232 articles passing the inclusion criteria.

**Results:**

Overall, we observed an increased median overall survival (OS, slope = 0.16 months per year, Pearson’s r = 0.23, *p* = 1.65e-44) for all study types. Nevertheless, reported survival outcomes varied by treatment modality. Only tumor treating fields (fitted median OS from 14.5 to 21.4 months, slope = 0.53 months per year) showed a stronger increase in positive outcomes than radiotherapy with concomitant and adjuvant temozolomide (fitted median overall survival from 12.0 to 20.0 months, slope = 0.31 months per year).

Despite making up only 5.6% of all studies, randomized controlled trials had the highest cumulative impact factor and citation count and US-based researchers dominated across all study types (33.9%). Women continued to be underrepresented, particularly as last authors (25.2% female first, 18.5% female last).

**Conclusion:**

The 75 years of reported survival of patients with glioblastoma document a slow increase in overall survival. While research by women as first authors shows an upward trend, gender inequality persists, particularly in the last authorship.

## Key points

- Survival of patients with glioblastoma has gradually increased over 75 years with lower survival in population-based studies.
- Among newer treatment approaches, only tumor treating fields is associated with increased survival.
- The glioblastoma research landscape is dominated by regional differences and a slowly diminishing authorship bias.

## Importance of the study

This 75-year comprehensive, bibliometric analysis indicates a slow but significant increase in reported overall patient survival. While standard chemoradiotherapy contributed to improved outcomes, tumor treating fields demonstrated a more substantial recent increase in reported survival benefits. Despite this progress, the field exhibits strong US-centered research and sex disparities in authorship persist, particularly in senior author roles.

## Introduction

Cancer patient outcomes are continually shaped by advancements in disease management and treatment, with oncology spearheading efforts in precision and preventive medicine. While patient survival considerably improved for many cancer types, partly in response to initiatives such as the cancer moonshot program [1], the outcome for patients with glioblastoma has remained stagnant over the last decades [2, 3]. Reported median overall survival (OS) ranges from 8–14 months at the population level [4] and from 20.9–48.1 months in phase 3 clinical trials [5, 6], where young age, methylated O^6^-methylguanine-DNA methyltransferase (*MGMT*) gene promoter, female sex and gross total resection have been established as important positive prognostic markers [7, 8].

Prior to 2005, radiation therapy was the only established treatment until the introduction of the Stupp protocol, i.e. radiotherapy with concurrent and adjuvant chemotherapy using temozolomide (TMZ), showed significant survival benefits [9]. This treatment remained the gold standard and was recently potentially refined with the addition of tumor treating fields (TTF), i.e. locally applied low-intensity, alternating electric fields with antimitotic effects [5]. Also, for patients with newly diagnosed glioblastoma with methylated *MGMT* promoter, the addition of lomustine to TMZ showed improved survival compared to the standard therapy [6]. Despite translational and clinical research efforts, targeted therapy with agents such as anti–vascular endothelial growth factor (anti-VEGF), integrins, programmed death-ligand 1 (PD-L1) checkpoint inhibitors, epidermal growth factor receptor (EGFR) antibody-drug conjugates or proteasome inhibitors were of limited success [10–14]. Their failure was largely ascribed to the molecular heterogeneity and transcriptional plasticity of glioblastomas [15]. Newer approaches currently under investigation include functional precision medicine, chimeric antigen receptor (CAR) -T cell therapy, tumor vaccines and oncolytic viruses [16, 17].

The longitudinal evolution of glioblastoma treatment and patient outcomes is documented by a body of literature that showed an exponential increase in articles starting from the 1990s, suggesting a growing research effort; however, previous work suggested a gender bias in oncology publications and medicine in general [18–21]. While authorship trends showed in general an increase in female first authorships in oncology and specifically in neuro-oncology [22], female last authorships still lagged considerably behind [18, 23].

Here, we map 75 years of glioblastoma research to track the evolution of overall survival across study types, treatment regimens and publication landscape. We then leveraged this dataset to examine sex disparities in authorship across time periods, types of study and geographic regions.

## Material and methods

### Search strategy and selection criteria

We conducted a manual search on PubMed.gov using the terms “glioblastoma AND overall survival”. The search strategy was restricted to publications from 1947–2022 that were conducted in humans and provided information on median overall survival (OS) during the period of studies. This resulted in 19,668 retrieved database entries.

Studies were selected according to the following inclusion criteria: 1) English language, 2) abstract availability, 3) full-text accessibility (open access, library access, Researchgate) and 3) reported median OS in the text from diagnosis using Kaplan Meier (KM) analysis. The following exclusion criteria were employed: 1) in vitro studies and animal studies, 2) studies in pediatric (< 18 years old) patients, 3) case reports, case series focusing on long-term survivors and reviews, 4) mixed malignant glioma groups, 5) brain stem glioma and thalamic glioma and 6) studies which reported survival data from KM analysis only in graphical format and reported median OS from the time of recurrence. This resulted in 2232 included studies, with 1,685,872 patients overall.

**Fig. 1:**
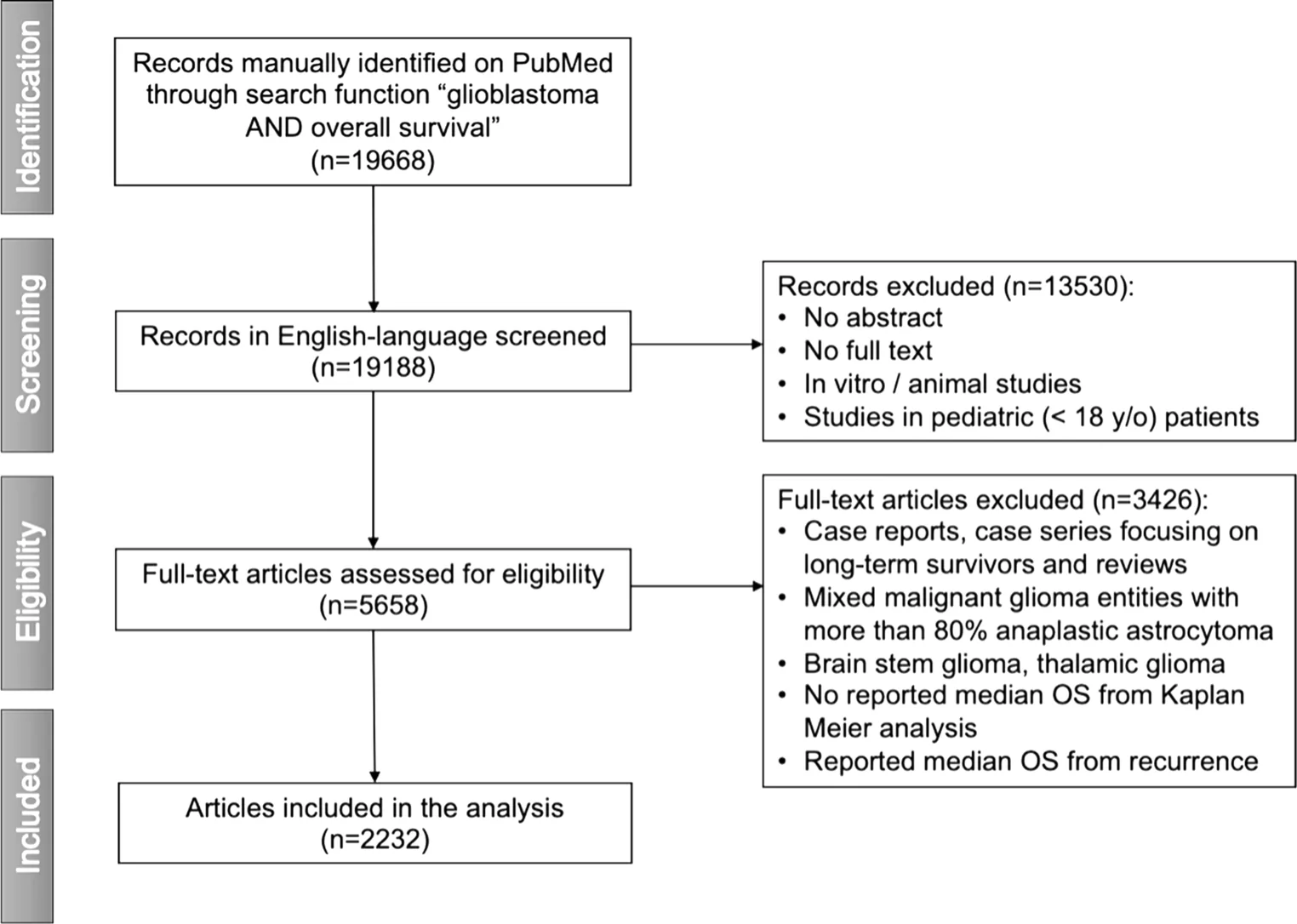
Flowchart presenting search strategy and study selection criteria. *OS* overall survival

A detailed workflow of the literature search is given in Fig. 1.

For every study, we collected the following metrics: number of patients, median OS time, recruitment period, type of study, year of publication, sex and affiliations of first and last author, journal, number of citations, and impact factors (IF) of the journal in 2022. Authorship positions and affiliations were assigned according to the order in the PubMed database. More specifically, the authors who were listed first on the list or whose names appeared first were referred to as “first authors,” and the authors whose names appeared last on the list were referred to as “last authors.” The genders of all authors were classified using the Python package gender-guesser (https://github.com/lead-ratings/gender-guesser), and ambiguities were manually double-checked and corrected if necessary. The type of study was categorized into case series (CS; not focusing on long-term survivors), clinical trial (CT), randomized controlled trial (RCT), meta-analysis and population-based study.

For cases without a reported recruitment period, the last 2, 5 and 8 years before the publication date were extrapolated according to sample size (< 20 patients: last 2 years, 20–50 patients: last 5 years, 50+: last 8 years).

The treatment regimen was classified into radiotherapy, chemotherapy (chloroethyl-nitrosoureas class (CNU), carmustine (BCNU), irinotecan, fotemustine, carboplatin, nimustin, vincristine, lomustine, prednimustine, cisplatin, procarbazine, teniposide, cediranib), temozolomide (TMZ), targeted therapy (tyrosine kinase inhibitors, i.e. apatinib, crizotinib, erlotinib, gefitinib, lapatinib, regorafenib), tumor treating fields, immunotherapy (peptide vaccines, checkpoint inhibitors, oncolytic viruses, autologous lymphoid effector cells specific against tumor-cells (ALECSAT)) and antiangiogenic treatment using bevacizumab.

The resulting list will be shared upon publication of the manuscript.

### Statistical analysis

Data were collected and entered in a Microsoft Excel sheet from 2020–2023 which was proofread between January and February 2024. Visualization and correlation analysis were performed with Python v3.11.5 (Python Software Foundation, Wilmington, DE, USA), using the packages scipy v1.11.4 and seaborn v0.13.2. Descriptive statistics were performed using SPSS v 25.0 (IBM Corp., Armonk, NY, USA).

## Results

### Overview of the included glioblastoma study landscape

Overall, 2232 articles were included in this study. The analysis consisted of 1462 case series (65.5%) with 314,866 patients, 534 clinical trials (23.9%) with 32,778 patients, 124 randomized controlled trials (5.6%) comprising 22,066 patients, 99 population-based studies (4.4%) with 1,303,190 patients and 13 meta-analyses (0.6%) with 12,972 patients published in 349 journals.

In addition, we analyzed studies including radiotherapy (*n* = 853, 38.2%) with a total of 311,370 patients, followed by chemotherapy (*n* = 316, 14.2%) with 61,601 patients, TMZ (*n* = 487, 21.8%) with 114,235 patients, targeted therapy (*n* = 21, 0.9%) with 1003 patients, tumor treating fields (*n* = 12, 0.5%) with 1602 patients, immunotherapy (*n* = 47, 2.1%) with 2700 patients, and bevacizumab (*n* = 131, 5.9%) with 38,168 patients.

### Continuous increase in median overall survival

**Fig. 2:**
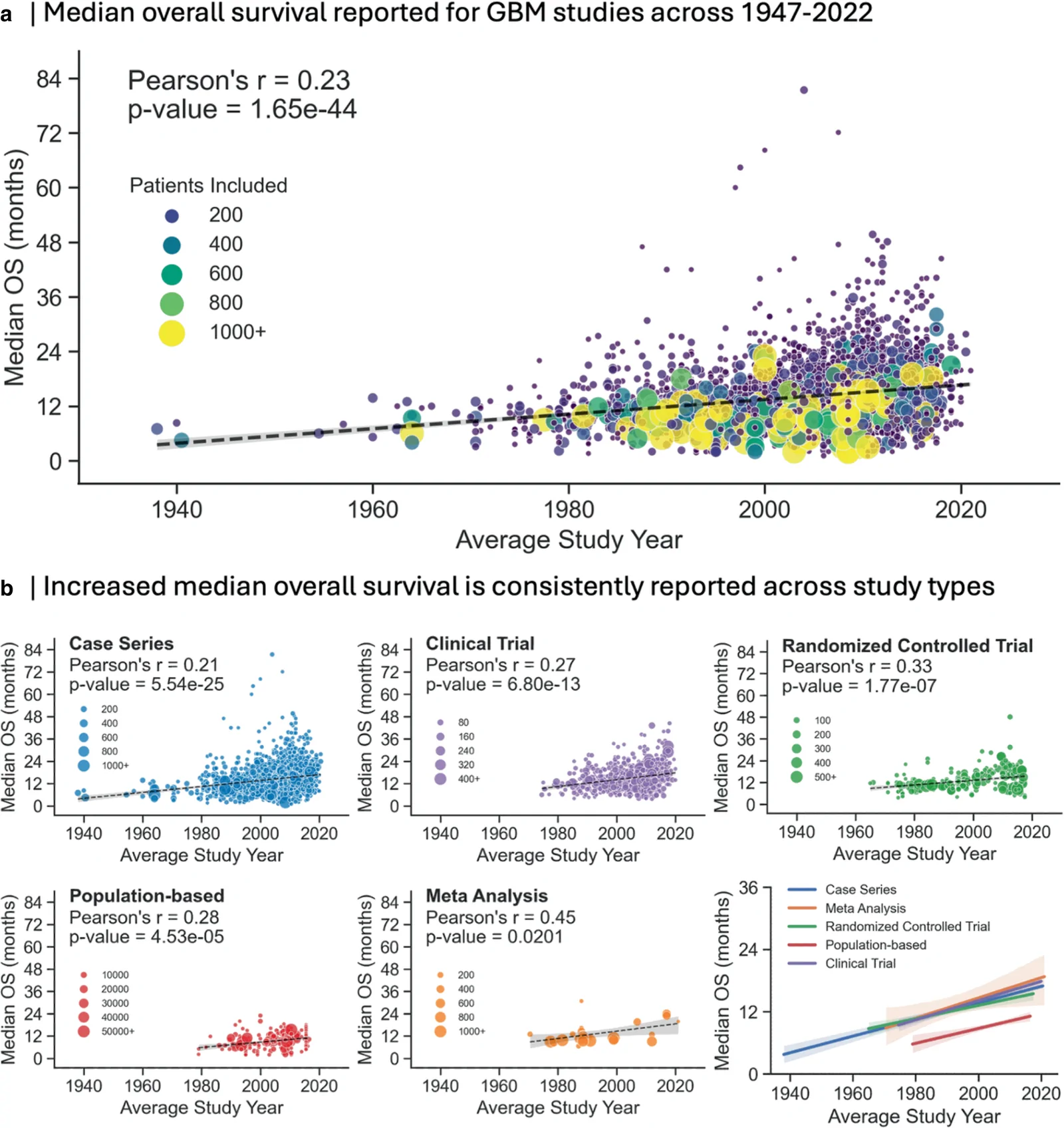
Median overall survival (OS) per average study year (mean of the recruitment period) from 1947 to 2022 and per study type. **a** Linear regression indicates a change in fitted values from 3.5 to 17.0 months median OS across all included glioblastoma studies, **b** an increase in median OS is found for all study types. Case series, clinical trials, randomized controlled trials, and meta-analysis reported comparable outcomes. Lower median OS was reported in population-based studies across the time period. *GBM* glioblastoma

Across all study types, there was a significant correlation between median overall survival and average study year (mean of the recruitment period) (slope = 0.16 months per year, Pearson’s r = 0.23, *p* = 1.65e-44) (Fig. 2a). This significant relationship was also found for each study type individually (Fig. 2b). Case series spanned the entire study time period of 1938–2021 and linear regression revealed an increase in fitted median OS from 3.8–17.1 months (slope = 0.16 months per year, Pearson’s r = 0.21, *p* = 5.54e-25). The included clinical trials, which were first reported in 1974 and showed an increase in fitted median OS from 9.4 to 18.0 months (slope = 0.19 months per year, Pearson’s r = 0.27, *p* = 6.80e-13). Randomized controlled trials, including studies from 1965, revealed a change in median OS from 8.7 to 15.6 months (slope = 0.13 months per year, Pearson’s r = 0.33, *p* = 1.77e-07). Meta-analyses were included from 1971 onwards and the reported median OS increased during the examined time period on the regression line from 8.9 to 18.9 months (slope = 0.20 months per year, Pearson’s r = 0.45, *p* = 0.0201). The lowest survival outcomes were reported in population-based studies, including from 1979 to 2017, with an increase in the fitted median OS from 5.7 to 11.3 months (slope = 0.14 months per year, Pearson’s r = 0.28, *p* = 4.53e-05).

Overall, case series, clinical trials, randomized controlled trials, and meta-analysis showed a similar trajectory of improvements in median OS, with comparable median OS endpoints. Population-based studies reported an overall lower improvement in patient survival outcomes.

### Treatment-dependent survival

**Fig. 3:**
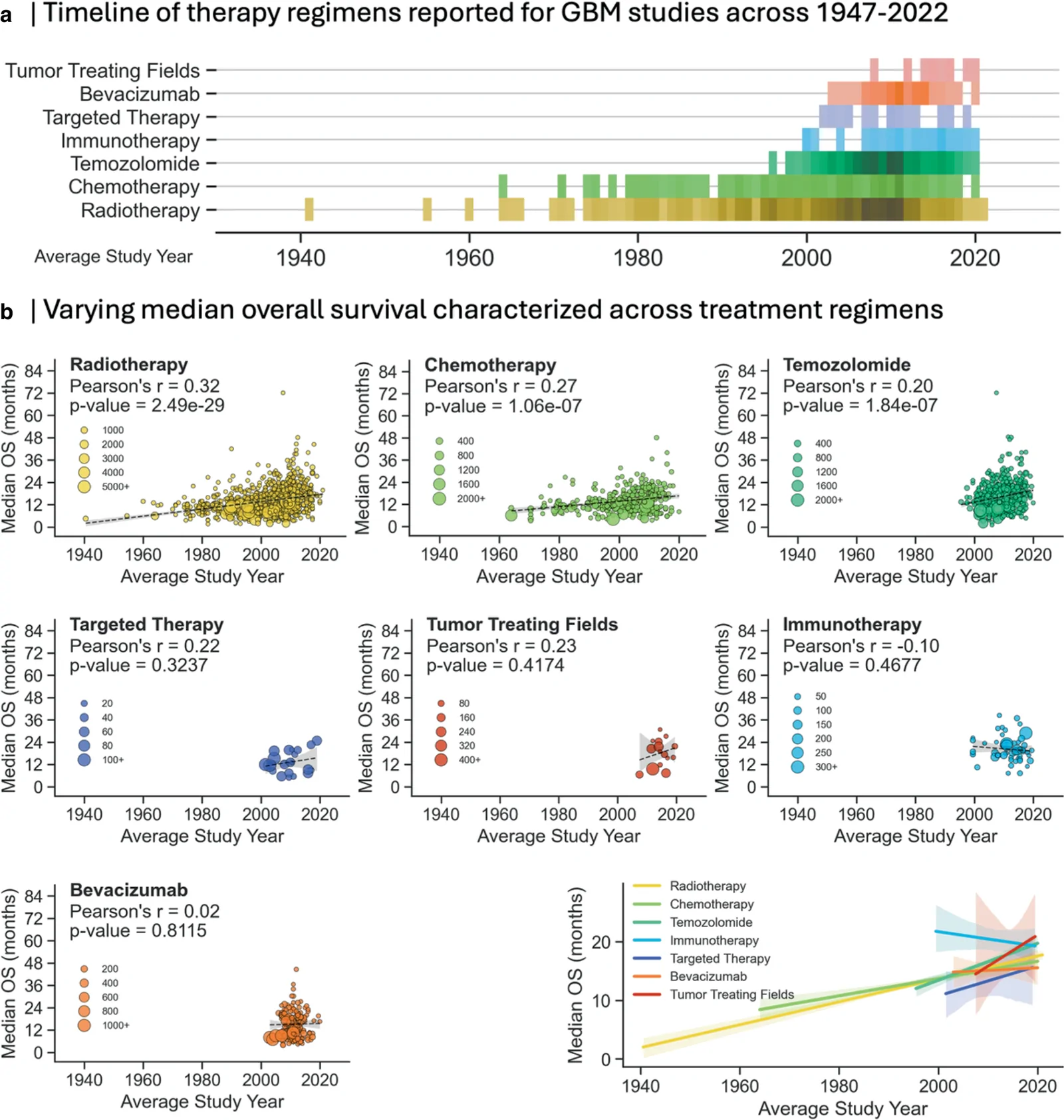
The evolution of glioblastoma therapy regimens across the time period from 1947 to 2022. The average study year represents the mean of the recruitment period. **a** The historical representation of the emergence of therapies over the time period 1947–2022, **b** an increased survival outcome is not reported for all treatment regimens. Overall, converging outcome endpoints were reported for all treatments. The steepest increase over the years was observed for tumor treating fields, while immunotherapy was the only treatment without a reported increase in median OS. *GBM* glioblastoma

Different glioblastoma treatment modalities across the first and second line have been reported over the examined time period. In the analyzed studies, radiotherapy was first reported in the 1940s, chemotherapy from the 1960s, and TMZ from the late 1990s prior to the advent of immunotherapies, targeted treatments, including bevacizumab and to the latest adoption of the tumor treating fields (Fig. 3a).

Not all treatment regimens show an increase in reported survival outcome (Fig. 3b). Studies reporting radiotherapy had the largest increase in median OS with fitted values on the regression line from 2.0 to 17.9 months (slope = 0.20 months per year, Pearson’s r = 0.32, *p* = 2.49e-29). Reported outcomes for chemotherapy showed a fitted median OS increase from 8.4 to 17.9 months (slope = 0.15 months per year, Pearson’s r = 0.27, *p* = 1.06e-07). A strong increase in median OS was reported for the use of TMZ in the treatment regimen with fitted outcome values increasing from 12.0 to 20.0 months (slope = 0.31 months per year, Pearson’s r = 0.20, *p* = 1.84e-07). No significant correlation between median OS and study year was found for publications reporting on targeted therapy (fitted OS 11.1–15.9, slope = 0.26 months per year, Pearson’s r = 0.22, *p* = 0.3237), immunotherapy (fitted OS 21.8–19.1, slope = −0.12 months per year, Pearson’s r = −0.10, *p* = 0.4677) and studies with bevacizumab (fitted OS 14.8–15.6, slope = 0.04 months per year, Pearson’s r = 0.02, *p* = 0.8115). Although no significant correlation between outcome and study year was found, treatment with tumor treating fields demonstrated the highest increase in survival (fitted OS 14.5–21.4, slope = 0.53 months per year, Pearson’s r = 0.23, *p* = 0.4174).

### Landscape of glioblastoma research across journals and geographic region

**Fig. 4:**
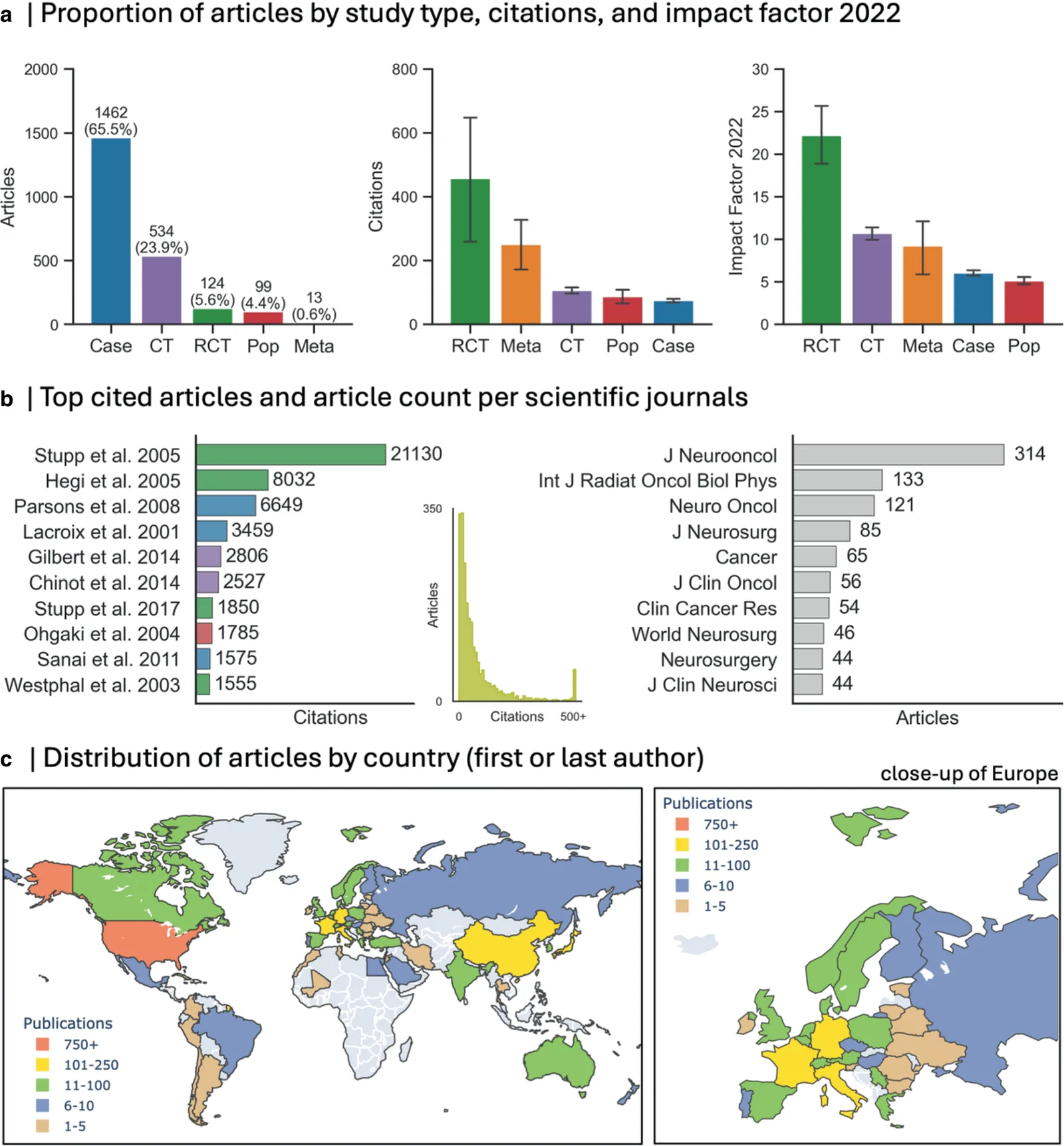
Overview of the glioblastoma publication landscape. **a** Proportion of articles by study type, citations, and impact factor 2022 indicates a high prevalence of case series but a larger impact of randomized controlled trials. Error bars denote standard error, **b** outliers were also observed for individual citations, and the majority of publications were cited less than 50 times in scientific journals, **c** world map of distribution of publications by country showing the dominance of the publication from the USA. *Case* case series, *CT* clinical trials, *RCT* randomized controlled trials, *Meta* meta-analysis, *Pop* population based studies

There was a divergence between the prevalence and the impact of articles reporting on different glioblastoma study types (Fig. 4a). Case series were by far the most reported study type in the investigated time period (*n* = 1462, 65.5%), followed by clinical trials (*n* = 534, 23.9%), randomized controlled trials (*n* = 124, 5.6%), population-based studies (*n* = 99, 4.4%), and meta-analyses (*n* = 13, 0.6%); however, the most citations were observed for randomized controlled trials with 457.5 citations on average. Meta-analyses were on average cited 250.8 times, clinical trials 106.8 times, and population-based studies 87.4 times. Case series were on average cited 75.2 times. Similarly, randomized controlled trials were published in high-impact journals (mean IF 22.2), followed by clinical trials (mean IF 10.7), meta-analyses (mean IF 9.2), case series (mean IF 6.1), and population-based studies (mean IF 5.1).

Outliers were also observed for individual citations and target journals (Fig. 4b). The most cited article (*n* = 21,130 citations) was the introduction of the Stupp protocol [9], which was followed by less than half of its citations (*n* = 8032) by the investigation of TMZ for patients with methylated *MGMT* promoter [24]. The majority of the published studies were cited less than 50 times (*n* = 1263, 56.6%), and only 58 studies (3.6%) were cited more than 500 times. By far the most studies were published in the Journal of Neuro-Oncology (*n* = 314, 14.1%).

Publications on glioblastoma research originated from 60 different countries (Fig. 4c); however, the research landscape was predominantly US-centered, with the USA accounting for more than 1/3 of publications (*n* = 756, 33.9%), followed by Germany (*n* = 237, 10.6%) and Japan (*n* = 158, 7.1%). Regions, such as Latin America (*n* = 24, 1.1%), Africa (*n* = 8, 0.36%), and Eastern Europe (*n* = 49, 2.2%) were underrepresented with significantly fewer contributions.

### Sex differences in authorship

**Fig. 5:**
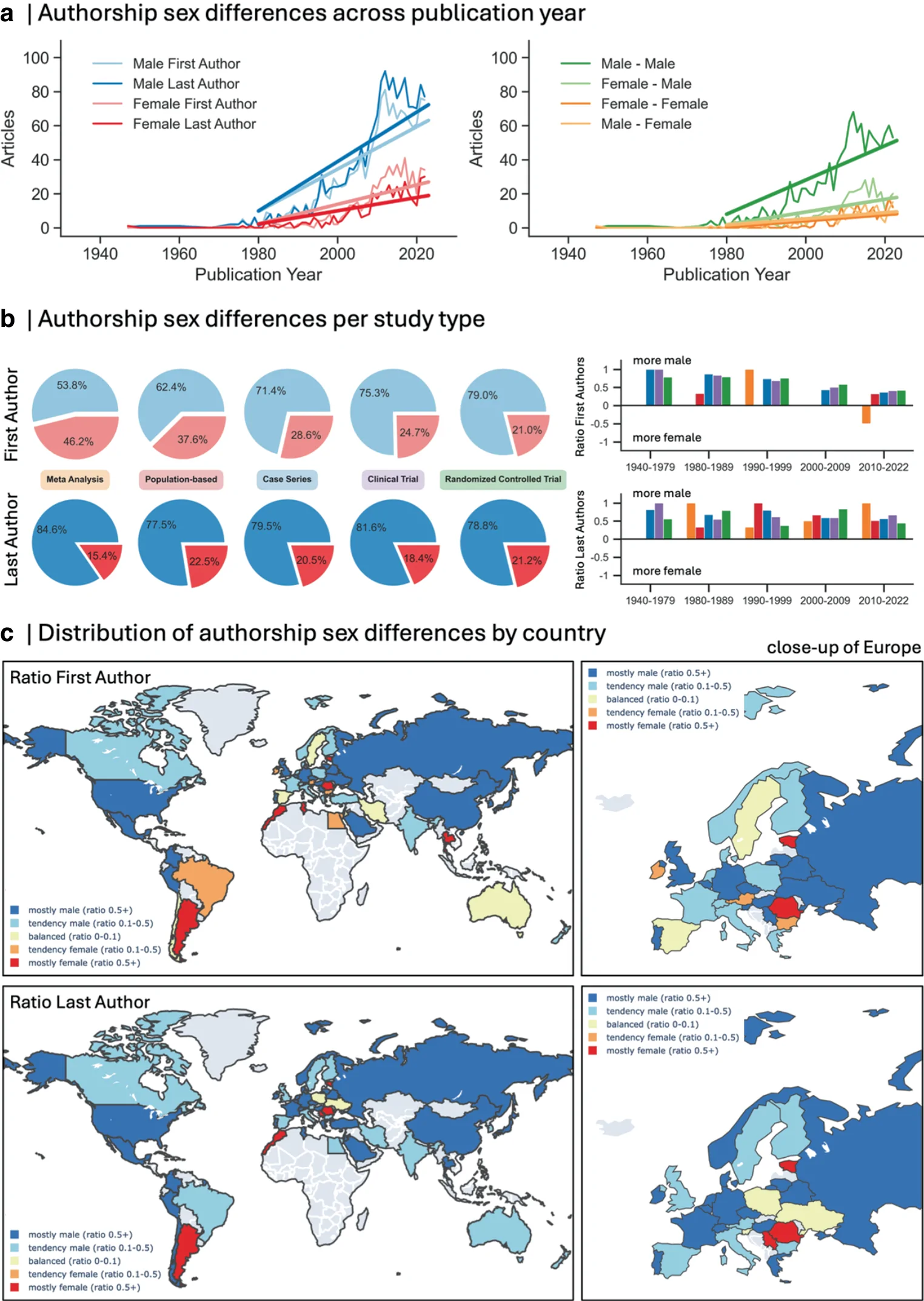
Authorship analysis by sex across publications. **a** Underrepresentation of women as first and last authors (25.2% female first, 18.5% female last). The steepest increase in authorship constellations was observed for male-dominated collaborations, particularly male first and male last authorships, **b** sex differences in first and last authorship per study type showing balanced ratio in first authors only for meta-analyses. A decreasing sex gap across study types and time only in the first authorship, **c** balanced ratio in first and last authorship in regions with lower publication rates

A sex disparity between authors was observed across the investigated time period (Fig. 5a). Overall, women were underrepresented as first and last authors (*n* = 561, 25.2% female first; *n* = 413, 18.5% female last vs. *n* = 1467, 65.8% male first; *n* = 1641, 73.5% male last) of the included articles. Fitting regression lines on the publications from 1980–2022 revealed diverging growth trajectories. The largest increase was observed for male last authors with fitted values from 9.9 to 72.3 publications (slope = 1.45 publications per year), followed by male first authors with an increase from 9.9 to 63.0 publications between 1980 and 2022 (slope = 1.24 publications per year). A diminished increase was observed for female authors, where female first authors showed a change in fitted values from 2.3 to 26.8 publications (slope = 0.57 publications per year), and for female last authors observed only a change from 2.1 to 18.9 publications (slope = 0.39 publications per year). When examining authorship constellations, the steepest increase by far was found for male first and male last authors (slope = 1.01 publications per year), with an increase from 7.9 to 51.2 publications. Female first and male last authorship constellations showed a larger increase (fitted values: 1.8 to 17.8 publications, slope = 0.37 publications per year) than male first and female last authors (fitted values: 1.6 to 9.4 publications, slope = 0.18 publications per year) and female first and female last author constellations (fitted values: 0.5 to 8.2 publications, slope = 0.18 publications per year).

Comparing authorship contributions per study type revealed that only the first authors of meta-analyses had an almost balanced sex distribution with 46.2% being female (Fig. 5b). The least female first authors (21.0%) were observed for randomized controlled trials. In contrast, the largest proportion of female last authors was 22.5% found in population-based studies, and the least was 18.4% in clinical trials. Sex disparities in first authorship decreased over time and for all study types but a clear narrowing of the sex gap in last authorship was not observed.

When considering countries of affiliation, a balanced ratio in male-female first authorship was achieved in Chile, Spain, Sweden, Iran, and Australia whereas in Slovenia, Poland, and Ukraine a balanced ratio in last authorship was observed. Regions with lower publication rates, such as Latin America, Africa, Southern and Eastern Europe, especially Argentina, Morocco, Romania, and Estonia were top performing in terms of female first and last authorship (Fig. 5c).

## Discussion

The present study provided a comprehensive overview of the landscape of almost 100 years of glioblastoma research. Overall, we found an increase in reported median OS across all study types but not all treatment regimens. At the same time, we documented a prevailing sex gap in authorship, with the disparity narrowing only slowly for first-author positions.

### Increase in overall survival

It was not surprising that the reported median OS was increasing over the years, consistent with observations across different cancer types [25]. We observed a similar outcome trend across all investigated study types with the exception of population-based studies, which consistently reported a lower median OS across the time period. Population-based studies are the most representative real-world settings and less prone to confounding factors [26], whereas clinical trials feature more homogeneous patient groups selecting younger patients with fewer comorbidities [27, 28]. Similarly, we observed the highest variability in survival rates in case series, which is likely due to the often small sample sizes and single-site nature. Intriguingly, considering the wealth of included publications, median survival rates were very similar across all study types except for population-based studies.

A previous study demonstrated that the survival rate of patients with glioblastoma did not improve from 1973 to 2001 [29]; however, starting from the twentieth century, an improvement in survival from 12.1 to 14.6 months was observed, mainly attributed to the landmark trial of radiotherapy with concomitant and adjuvant TMZ [9]. Indeed, our findings confirmed an increase in reported outcomes associated with such treatment, in line with results from other studies [30, 31]. Notably, regression analysis revealed an even stronger increase (steeper slope) in reported outcomes for tumor treating fields (TTF) (slope = 0.53 months per year) compared to other modalities; however, this finding must be interpreted with caution. This category represents only 0.5% of the total dataset (*n* = 12 studies) and is concentrated within the most recent study window. The limited sample size likely contributed to the lack of statistical significance for this correlation (*p* = 0.4174). Furthermore, the numerically higher slope may be influenced by publication enthusiasm or selection bias common in the early adoption phases of a new technology [32]. Therefore, while the trend is promising, the comparative magnitude of the TTF effect relative to long-standing treatments such as radiotherapy or TMZ remains to be validated as more longitudinal data become available.

In contrast, no increase in overall survival was found for immunotherapies and bevacizumab. In the context of antiangiogenic treatment, it is important to note that we did not include progression-free survival as a study endpoint, which was the main finding from bevazicumab [33].

Likewise, it is believed that immunotherapies possess enormous potential for treating glioblastoma but to date the majority of studies primarily revolved around immune checkpoint inhibitors whose effectiveness failed for the majority of patients with glioblastoma so far [34]; however, newer approaches embracing cancer vaccines, intratumoral oncolytic viral treatment or adoptive cell therapy, such as chimeric antigen receptor (CAR) T cells have shown promising results so far [35–38].

### The landscape of glioblastoma research

The landscape of glioblastoma research significantly evolved, with contributions from researchers worldwide publishing a wide range of studies in diverse journals. There has been a significant rise in studies on prognosis, patient survival and treatments for glioblastoma from 1973 to December 2022 based on previous bibliometric analyses [39, 40]. In our study, although case series were the most published study type overall, randomized controlled trials showed the greatest impact in terms of citation counts and high-impact publications. Starting from the 2000s, RCTs became the pinnacle of clinical research, resulting in a correlation between the number of RCTs published in a journal and its overall impact factor [41]. The second most cited study type was meta-analyses, which typically seek to provide evidence-based recommendations, increase the generalizability of research findings, and close research gaps.

Our findings confirmed a regional disparity in glioblastoma research, indicating a gap between the USA and the rest of the world, demonstrating the importance of accessible funding resources, which are 4 times higher in the USA than in Europe [42]. The findings reinforce the need for research support and collaboration between countries.

### Gender disparities in authorship

When looking at differences in the authorship representation, we observed that females were in the minority for both the first and last authorship positions. This is in line with previous studies that showed an underrepresentation of women in many oncology journals, particularly as senior authors [18, 43, 44]. On a positive note, we observed an increased female-to-male ratio in first authorship in the last two decades, which is in line with a recent study that showed a general increase in female authorships in neuro-oncology [22]; however, our results showed that females remained underrepresented especially among the last authors. A gender parity seemed to be reached in the first authorship only in meta-analyses. Notably, in randomized controlled trials, the study type with the greatest publication impact, the ratio of female first authors was the lowest.

Despite the ongoing male domination of publications in western countries, regions that generally have lower publication rates, i.e. Latin America, Africa, and Southern and Eastern Europe, achieved a more equitable representation of male and female authors.

### Strengths and limitations

The strengths of this study included a comprehensive dataset of 2232 articles from 349 scientific journals spanning over nearly a century long timeframe and thus incorporating diverse perspectives. To our knowledge, this is the first study of its kind including manually curated data encompassing 75 years to examine the reported survival of glioblastoma patients across different study types and treatment options, synthesizing a valuable summary and informing clinical practices. It also sheds light on publication bias by study type, gender, and geographic region. Manual screening of publications surpasses software tools by allowing a thorough evaluation of quality, relevance and methodological rigor, which software, limited by metadata and complex language, cannot yet achieve. By excluding reviews, we focused on primary research for a more accurate assessment of research trends, individual researchers and journals that contribute to novel research and to avoid the bias of synthesizing secondary data.

Our study has several limitations. First, the data were obtained solely from PubMed and were restricted to English-language articles. Secondly, we included only articles that clearly reported the median OS from diagnosis to death in contrast to from time of recurrence to death. We extracted only results from KM analysis in text, and no plot-digitizer tool was used to infer outcomes from figures; however, while potentially missing some reported outcomes, this implied that we only included reliable median OS. Our findings may reflect geographic skewing due to the overrepresentation of studies originating from high-income countries, leading to a potential overestimation of global survival outcomes. Also, tumor classification (i.e. primary and secondary glioblastoma) remained constant for the majority of our study period with a major change in paradigm only from 2021 onwards (i.e. glioblastoma IDH wild type versus astrocytoma IDH mutant) [45]. Only full-text articles were included in this analysis introducing a bias towards recently published articles. The inability to confirm the uniqueness of all enrolled patients is bound to the limitations of primary studies, acknowledging patient overlap as a common challenge. By extracting the recruitment period, sample size and study design for each publication and by differentiating case series, randomized controlled trials, clinical trials, population-based studies and meta-analyses, we attempted to reduce obvious overlap; however, a complete elimination of patient duplication was not feasible, as e.g. large datasets, such as the Surveillance, Epidemiology, and End Results (SEER) studies, are frequently reused in retrospective studies, causing overlap. Nevertheless, given the long study period and focus on aggregate trends across publications, any cohort overlap is unlikely to distort the observed survival trajectories.

We included information on treatment modalities without further stratification into first line versus further lines of treatments as this information was not widely available from older literature and certain article types such as population-based studies. Future studies would benefit from more granular information, especially in the context of specific drugs such as bevacizumab. Furthermore, citation counts depend on publication time, with older publications being able to amass more citations. It was not always possible to fully ascertain the full name and sex of the authors. Although Python package gender-guesser was used, manual curation led to changes in 7.2% cases to correct errors, and 16.8% of authors remained indeterminate, predominantly in Asia where gender-neutral given names are common. For indeterminate cases, authors were excluded from the analyses.

## Conclusion

In conclusion, the past century of glioblastoma research resulted in a modest increase in survival that was reported across all study types. While different treatment regimens emerged over the years, not all reported an increase in overall survival, and only tumor treatingf showed a stronger increase in positive outcomes than radiotherapy with concomitant and adjuvant temozolomide. Additionally, our findings support the need to achieve sex parity in leadership roles within glioblastoma studies, crucial for driving innovation and accelerating discoveries.

## References

[1] Shiels MS, Lipkowitz S, Campos NG, Schiffman M, Schiller JT, Freedman ND, et al. Opportunities for achieving the Cancer Moonshot goal of a 50 % reduction in cancer mortality by 2047. Cancer Discov. 2023;13(5):1084–99. 37067240 10.1158/2159-8290.CD-23-0208 PMC10164123

[2] Tykocki T, Eltayeb M. Ten-year survival in glioblastoma A systematic review. J Clin Neurosci. 2018;54:7–13. 29801989 10.1016/j.jocn.2018.05.002

[3] Tan AC, Ashley DM, López GY, Malinzak M, Friedman HS, Khasraw M. Management of glioblastoma: State of the art and future directions. CA Cancer J Clin. 2020;70(4):299–312. 32478924 10.3322/caac.21613

[4] Ostrom QT, Price M, Neff C, Cioffi G, Waite KA, Kruchko C, et al. CBTRUS Statistical Report: Primary Brain and Other Central Nervous System Tumors Diagnosed in the United States in 2016-2020. Neuro Oncol. 2023;25(12 Suppl 2):iv1–99. 37793125 10.1093/neuonc/noad149 PMC10550277

[5] Stupp R, Taillibert S, Kanner A, Read W, Steinberg D, Lhermitte B, et al. Effect of Tumor-Treating Fields Plus Maintenance Temozolomide vs Maintenance Temozolomide Alone on Survival in Patients With Glioblastoma: A Randomized Clinical Trial. JAMA. 2017;318(23):2306–16. 29260225 10.1001/jama.2017.18718 PMC5820703

[6] Herrlinger U, Tzaridis T, Mack F, Steinbach JP, Schlegel U, Sabel M, et al. Lomustine-temozolomide combination therapy versus standard temozolomide therapy in patients with newly diagnosed glioblastoma with methylated MGMT promoter (CeTeG/NOA-09): a randomised, open-label, phase 3 trial. Lancet [Internet]. 2019;393(10172):678–88. 30782343 10.1016/S0140-6736(18)31791-4

[7] Franceschi E, Tosoni A, Minichillo S, Depenni R, Paccapelo A, Bartolini S, et al. The Prognostic Roles of Gender and O6-Methylguanine-DNA Methyltransferase Methylation Status in Glioblastoma Patients: The Female Power. World Neurosurg. 2018;112:e342–e7. 29337169 10.1016/j.wneu.2018.01.045

[8] Tian M, Ma W, Chen Y, Yu Y, Zhu D, Shi J, et al. Impact of gender on the survival of patients with glioblastoma. Biosci Rep [Internet]. 2018 Dec 21;38(6). 10.1042/BSR20180752 PMC6239255 30305382

[9] Stupp R, Mason WP, van den Bent MJ, Weller M, Fisher B, Taphoorn MJB, et al. Radiotherapy plus concomitant and adjuvant temozolomide for glioblastoma. N Engl J Med. 2005;352(10):987–96. 15758009 10.1056/NEJMoa043330

[10] Gilbert MR, Dignam JJ, Armstrong TS, Wefel JS, Blumenthal DT, Vogelbaum MA, et al. A randomized trial of bevacizumab for newly diagnosed glioblastoma. N Engl J Med. 2014;370(8):699–708. 24552317 10.1056/NEJMoa1308573 PMC4201043

[11] Weller M, Butowski N, Tran DD, Recht LD, Lim M, Hirte H, et al. Rindopepimut with temozolomide for patients with newly diagnosed, EGFRvIII-expressing glioblastoma (ACT IV): a randomised, double-blind, international phase 3 trial. Lancet Oncol [Internet]. 2017;18(10):1373–85. 28844499 10.1016/S1470-2045(17)30517-X

[12] Reardon DA, Brandes AA, Omuro A, Mulholland P, Lim M, Wick A, et al. Effect of Nivolumab vs Bevacizumab in Patients With Recurrent Glioblastoma: The CheckMate 143 Phase 3 Randomized Clinical Trial. JAMA Oncol [Internet]. 2020;6(7):1003–10. 32437507 10.1001/jamaoncol.2020.1024 PMC7243167

[13] Lassman AB, Pugh SL, Wang TJC, Aldape K, Gan HK, Preusser M, et al. Depatuxizumab mafodotin in EGFR-amplified newly diagnosed glioblastoma: A phase III randomized clinical trial. Neuro Oncol. 2023;25(2):339–50. 35849035 10.1093/neuonc/noac173 PMC9925712

[14] Roth P, Gorlia T, Reijneveld JC, de Vos F, Idbaih A, Frenel JS, et al. Marizomib for patients with newly diagnosed glioblastoma: A randomized phase 3 trial. Neuro Oncol. 2024;26(9):1670–82. 38502052 10.1093/neuonc/noae053 PMC11376448

[15] Le Rhun E, Preusser M, Roth P, Reardon DA, van den Bent M, Wen P, et al. Molecular targeted therapy of glioblastoma. Cancer Treat Rev. 2019;80:101896. 31541850 10.1016/j.ctrv.2019.101896

[16] Todo T, Ino Y, Ohtsu H, Shibahara J, Tanaka M. A phase I/II study of triple-mutated oncolytic herpes virus G47∆ in patients with progressive glioblastoma. Nat Commun. 2022;13(1)4119. 35864115 10.1038/s41467-022-31262-y PMC9304402

[17] Luksik AS, Yazigi E, Shah P, Jackson CM. CAR T Cell Therapy in Glioblastoma: Overcoming Challenges Related to Antigen Expression. Cancers [Internet]. 2023 Feb 23;15(5). 10.3390/cancers15051414 PMC10000604 36900205

[18] Dalal NH, Chino F, Williamson H, Beasley GM, Salama AKS, Palta M. Mind the gap: Gendered publication trends in oncology. Cancer. 2020;126(12):2859–65. 32212334 10.1002/cncr.32818

[19] Sidhu R, Rajashekhar P, Lavin VL, Parry J, Attwood J, Holdcroft A, et al. The gender imbalance in academic medicine: a study of female authorship in the United Kingdom. J R Soc Med. 2009;102(8):337–42. 19679736 10.1258/jrsm.2009.080378 PMC2726808

[20] P C, RM W. Gender Disparity in Citations in High-Impact Journal Articles. JAMA Netw Open. 2021;4(7)e2114509. 34213560 10.1001/jamanetworkopen.2021.14509 PMC8254129

[21] Brück O. A bibliometric analysis of the gender gap in the authorship of leading medical journals. Communications Medicine [Internet]. 2023 [cited 2024 Oct 10];3. 10.1038/s43856-023-00417-3 PMC10713825 38082119

[22] Behmer Hansen RT, Behmer Hansen RA, Gold JL, Batchu S, Lozada RD, Palma SD, et al. Neuro-oncology authorship trends in gender since 1944: a systematic review of 14,020 articles from five top-tier academic journals. J Neurosurg [Internet]. 2023;139(1):1–10. 36433875 10.3171/2022.10.JNS221183

[23] Berghoff AS, Sessa C, Yang JCH, Tsourti Z, Tsang J, Tabernero J, et al. Female leadership in oncology-has progress stalled? Data from the ESMO W4O authorship and monitoring studies. ESMO Open. 2021;6(6)100281. 34924143 10.1016/j.esmoop.2021.100281 PMC8710465

[24] Hegi ME, Diserens AC, Gorlia T, Hamou MF, de Tribolet N, Weller M, et al. MGMT gene silencing and benefit from temozolomide in glioblastoma. N Engl J Med [Internet]. 2005 Mar 10 [cited 2024 Oct 3];352(10). 10.1056/NEJMoa043331 15758010

[25] Miller KD, Nogueira L, Devasia T, Mariotto AB, K Yabroff R, Jemal A, et al. Cancer treatment and survivorship statistics, 2022. CA Cancer J Clin. 2022;72(5):409–36. 35736631 10.3322/caac.21731

[26] Pulte D, Weberpals J, Jansen L, Brenner H. Changes in population-level survival for advanced solid malignancies with new treatment options in the second decade of the 21st century. Cancer. 2019;125(15):2656–65. 31095726 10.1002/cncr.32160

[27] Shahar T, Nossek E, Steinberg DM, Rozovski U, Blumenthal DT, Bokstein F, et al. The impact of enrollment in clinical trials on survival of patients with glioblastoma. J Clin Neurosci [Internet]. 2012;19(11):1530–4. 22989795 10.1016/j.jocn.2012.04.005

[28] Skaga E, Skretteberg MA, Johannesen TB, Brandal P, Vik-Mo EO, Helseth E, et al. Real-world validity of randomized controlled phase III trials in newly diagnosed glioblastoma: to whom do the results of the trials apply? Neurooncol Adv [Internet]. 2021;3(1)vdab008. 33665615 10.1093/noajnl/vdab008 PMC7914075

[29] Deorah S, Lynch CF, Sibenaller ZA, Ryken TC. Trends in brain cancer incidence and survival in the United States: Surveillance, Epidemiology, and End Results Program, 1973 to 2001. Neurosurg Focus. 2006;20(4)E1. 16709014 10.3171/foc.2006.20.4.E1

[30] Johnson DR, O’Neill BP. Glioblastoma survival in the United States before and during the temozolomide era. J Neurooncol [Internet]. 2012;107(2):359–64. 22045118 10.1007/s11060-011-0749-4

[31] Eriksson M, Kahari J, Vestman A, Hallmans M, Johansson M, Bergenheim AT, et al. Improved treatment of glioblastoma - changes in survival over two decades at a single regional Centre. Acta Oncol [Internet]. 2019;58(3):334–41. 30732527 10.1080/0284186X.2019.1571278

[32] Du X, Chen C, Xiao Y, Cui Y, Yang L, Li X, et al. Research on application of tumor treating fields in glioblastoma: A bibliometric and visual analysis. Front Oncol. 2022;12:1055366. 36439464 10.3389/fonc.2022.1055366 PMC9684468

[33] Wick W, Gorlia T, Bendszus M, Taphoorn M, Sahm F, Harting I, et al. Lomustine and Bevacizumab in Progressive Glioblastoma. N Engl J Med. 2017;377(20):1954–63. 29141164 10.1056/NEJMoa1707358

[34] Sampson JH, Gunn MD, Fecci PE, Ashley DM. Brain immunology and immunotherapy in brain tumours. Nat Rev Cancer. 2020;20(1):12–25. 31806885 10.1038/s41568-019-0224-7 PMC7327710

[35] Todo T, Ito H, Ino Y, Ohtsu H, Ota Y, Shibahara J, et al. Intratumoral oncolytic herpes virus G47∆ for residual or recurrent glioblastoma: a phase 2 trial. Nat Med. 2022;28(8):1630–9. 35864254 10.1038/s41591-022-01897-x PMC9388376

[36] Bagley SJ, Logun M, Fraietta JA, Wang X, Desai AS, Bagley LJ, et al. Intrathecal bivalent CAR T cells targeting EGFR and IL13Rα2 in recurrent glioblastoma: phase 1 trial interim results. Nat Med. 2024;30(5):1320–9. 38480922 10.1038/s41591-024-02893-z PMC13123313

[37] Choi BD, Gerstner ER, Frigault MJ, Leick MB, Mount CW, Balaj L, et al. Intraventricular CARv3-TEAM‑E T Cells in Recurrent Glioblastoma. N Engl J Med [Internet]. 2024 Apr 11 [cited 2024 Oct 11]; 10.1056/NEJMoa2314390 PMC11162836 38477966

[38] Lv K, Du X, Chen C, Yu Y. Research hotspots and trend of glioblastoma immunotherapy: a bibliometric and visual analysis. Front Oncol. 2024;14:1361530. 39175478 10.3389/fonc.2024.1361530 PMC11339877

[39] Montes-Escobar K, de la Hoz MJ, Castillo-Cordova P, Duran-Ospina JP, Bravo-Saltos RK, Lapo-Talledo GJ, et al. Glioblastoma: a comprehensive approach combining bibliometric analysis, Latent Dirichlet Allocation, and HJ-Biplot : Glioblastoma insights and trends: a 49-year bibliometric analysis. Neurosurg Rev. 2024;47(1)209. 38724684 10.1007/s10143-024-02440-x

[40] Alirezaei Z, Amouheidari A, BasirianJahromi R, Seyyedhosseini S, Hamidi A. Survival analysis of glioblastoma: A scientometric perspective. World Neurosurg. 2025;194(123476)123476. 39577630 10.1016/j.wneu.2024.11.059

[41] Weale AR, Lear PA. Randomised controlled trials and quality of journals. Lancet. 2003;361(9370):1749–50. 12767778 10.1016/s0140-6736(03)13360-0

[42] Sobocki P, Lekander I, Berwick S, Olesen J, Jönsson B. Resource allocation to brain research in Europe (RABRE). Eur J Neurosci. 2006;24(10):2691–3. 17156195 10.1111/j.1460-9568.2006.05116.x

[43] Filardo G, da Graca B, Sass DM, Pollock BD, Smith EB, Martinez MAM. Trends and comparison of female first authorship in high impact medical journals: observational study (1994-2014). BMJ [Internet]. 2016 Mar 2 [cited 2024 Sep 19];352. 10.1136/bmj.i847 PMC4775869 26935100

[44] Hornstein P, Tuyishime H, Mutebi M, Lasebikan N, Rubagumya F, Fadelu T. Authorship Equity and Gender Representation in Global Oncology Publications. JCO Glob Oncol. 2022;8:e2100369. 35015565 10.1200/GO.21.00369 PMC8769149

[45] Louis DN, Perry A, Wesseling P, Brat DJ, Cree IA, Figarella-Branger D, et al. The 2021 WHO Classification of Tumors of the Central Nervous System: a summary. Neuro Oncol. 2021;23(8):1231–51. 34185076 10.1093/neuonc/noab106 PMC8328013

